# Evaluating the factor structure and measurement invariance of the 20-item short version of the UPPS-P Impulsive Behavior Scale across multiple countries, languages, and gender identities

**DOI:** 10.1177/10731911241259560

**Published:** 2024-07-26

**Authors:** Loïs Fournier, Beáta Bőthe, Zsolt Demetrovics, Mónika Koós, Shane W. Kraus, Léna Nagy, Marc N. Potenza, Rafael Ballester-Arnal, Dominik Batthyány, Sophie Bergeron, Peer Briken, Julius Burkauskas, Georgina Cárdenas-López, Joana Carvalho, Jesús Castro-Calvo, Lijun Chen, Giacomo Ciocca, Ornella Corazza, Rita I. Csako, David P. Fernandez, Hironobu Fujiwara, Elaine F. Fernandez, Johannes Fuss, Roman Gabrhelík, Ateret Gewirtz-Meydan, Biljana Gjoneska, Mateusz Gola, Joshua B. Grubbs, Hashim T. Hashim, Md. Saiful Islam, Mustafa Ismail, Martha C. Jiménez-Martínez, Tanja Jurin, Ondrej Kalina, Verena Klein, András Költő, Sang-Kyu Lee, Karol Lewczuk, Chung-Ying Lin, Christine Lochner, Silvia López-Alvarado, Kateřina Lukavská, Percy Mayta-Tristán, Dan J. Miller, Oľga Orosová, Gábor Orosz, Fernando P. Ponce, Gonzalo R. Quintana, Gabriel C. Quintero Garzola, Sungkyunkwan University Research Team, Jano Ramos-Diaz, Kévin Rigaud, Ann Rousseau, Marco De Tubino Scanavino, Marion K. Schulmeyer, Pratap Sharan, Mami Shibata, Sheikh Shoib, Vera Sigre-Leirós, Luke Sniewski, Ognen Spasovski, Vesta Steibliene, Dan J. Stein, Julian Strizek, Meng-Che Tsai, Berk C. Ünsal, Marie-Pier Vaillancourt-Morel, Marie Claire Van Hout, Joël Billieux

**Affiliations:** 1 Institute of Psychology, University of Lausanne, Lausanne, Switzerland; 2 Département de Psychologie, Université de Montréal, Montréal, Canada; 3 Institute of Psychology, ELTE Eötvös Loránd University, Budapest, Hungary; 4 Centre of Excellence in Responsible Gaming, University of Gibraltar, Gibraltar, Gibraltar; 5 Department of Psychology, University of Nevada, Las Vegas, Las Vegas, Nevada, United States of America; 6 Yale University School of Medicine, New Haven, Connecticut, United States of America; 7 Connecticut Council on Problem Gambling, Wethersfield, Connecticut, United States of America; 8 Connecticut Mental Health Center, New Haven, Connecticut, United States of America; 9 Departamento de Psicología Básica, Clínica y Psicobiología, University Jaume I of Castellón, Spain; 10 Institute for Behavioural Addictions, Sigmund Freud University Vienna, Austria; 11 Institute for Sex Research, Sexual Medicine, and Forensic Psychiatry, University Medical Centre Hamburg-Eppendorf, Hamburg, Germany; 12 Laboratory of Behavioral Medicine, Neuroscience Institute, Lithuanian University of Health Sciences, Palanga, Lithuania; 13 Virtual Teaching and Cyberpsychology Laboratory, School of Psychology, National Autonomous University of Mexico, Mexico; 14 William James Center for Research, Departamento de Educação e Psicologia, Universidade de Aveiro, Aveiro, Portugal; 15 Department of Personality, Assessment, and Psychological Treatments, University of Valencia, Spain; 16 Department of Psychology, College of Humanity and Social Science, Fuzhou University, China; 17 Section of Sexual Psychopathology, Department of Dynamic and Clinical Psychology, and Health Studies, Sapienza University of Rome, Rome, Italy; 18 Department of Clinical, Pharmaceutical and Biological Sciences, University of Hertfordshire, United Kingdom; 19 Department of Psychology and Cognitive Science, University of Trento, Italy; 20 Auckland University of Technology, New Zealand; 21 Nottingham Trent University, United Kingdom; 22 Department of Neuropsychiatry, Graduate School of Medicine, Kyoto University, Kyoto, Japan; 23 Decentralized Big Data Team, RIKEN Center for Advanced Intelligence Project, Tokyo, Japan; 24 The General Research Division, Osaka University Research Center on Ethical, Legal and Social Issues, Osaka, Japan; 25 Higher Education Learning Philosophy University, Kuala Lumpur, Malaysia; 26 Institute of Forensic Psychiatry and Sex Research, Center for Translational Neuro- and Behavioral Sciences, University of Duisburg-Essen, Essen, Germany; 27 Department of Addictology, First Faculty of Medicine, Charles University, Prague, Czech Republic; 28 Department of Addictology, General University Hospital in Prague, Prague, Czech Republic; 29 School of Social Work, Faculty of Social Welfare and Health Sciences, University of Haifa, Israel; 30 Macedonian Academy of Sciences and Arts, Skopje, Republic of North Macedonia; 31 Institute of Psychology, The Polish Academy of Sciences, Warszawa, Poland; 32 Institute for Neural Computations, University of California San Diego, United States of America; 33 Center on Alcohol, Substance use, And Addictions, The University of New Mexico, Albuquerque, United States of America; 34 College of Medicine, University of Baghdad, Iraq; 35 College of Medicine, University of Warith Al-Anbiyaa, Karbala, Iraq; 36 Department of Public Health and Informatics, Jahangirnagar University, Dhaka, Bangladesh; 37 Centre for Advanced Research Excellence in Public Health, Dhaka, Bangladesh; 38 Universidad Pedagógica y Tecnológica de Colombia, Tunja, Colombia; 39 Grupo de Investigación Biomédica y de Patología, Tunja, Colombia; 40 Grupo Medición y Evaluación Psicológica en Contextos Básicos y Aplicados, Tunja, Colombia; 41 Department of Psychology, Humanities and Social Sciences, University of Zagreb, Croatia; 42 Department of Educational Psychology and Psychology of Health, Pavol Jozef Safarik University in Kosice, Slovakia; 43 School of Psychology, University of Southampton, United Kingdom; 44 Health Promotion Research Centre, University of Galway, Ireland; 45 Department of Psychiatry, Hallym University Chuncheon Sacred Heart Hospital, South Korea; 46 Chuncheon Addiction Management Center, South Korea; 47 Institute of Psychology, Cardinal Stefan Wyszynski University, Warsaw, Poland; 48 College of Medicine, National Cheng Kung University, Tainan, Taiwan; 49 SAMRC Unit on Risk & Resilience in Mental Disorders, Stellenbosch University, South Africa; 50 University of Cuenca, Ecuador; 51 Facultad de Medicina, Universidad Científica del Sur, Lima, Perú; 52 College of Healthcare Sciences, James Cook University, Townsville, Queensland, Australia; 53 Artois University, Arras, France; 54 Facultad de Psicología, Universidad de Talca, Chile; 55 Departamento de Psicología y Filosofía, Facultad de Ciencias Sociales, Universidad de Tarapacá, Arica, Arica y Parinacota, Chile; 56 Florida State University ? Republic of Panama, Ciudad del Saber, Republic of Panama; 57 Sistema Nacional de Investigación, Secretaría Nacional de Ciencia, Tecnología e Innovación, Ciudad del Saber, Republic of Panama; 58 Department of Psychology, Sungkyunkwan University, South Korea; 59 Facultad de Ciencias de la Salud, Universidad Privada del Norte, Lima, Perú; 60 Leuven School for Mass Communication, Katholieke Universiteit Leuven, Belgium; 61 Department of Psychiatry, Schulich School of Medicine & Dentistry, Western University, London, Ontario, Canada; 62 Lawson Health Research Institute, London, Ontario, Canada; 63 Departmento e Instituto de Psiquiatria, Hospital das Clinicas and Experimental Pathophysiology Post Graduation Program, Faculdade de Medicina, Universidade de São Paulo, Brazil; 64 Universidad Privada de Santa Cruz de la Sierra, Bolivia; 65 Department of Psychiatry, All India Institute of Medical Sciences, New Delhi, India; 66 Department of Health Services, Srinagar, India; 67 Sharda University, Greater Noida, India; 68 Psychosis Research Centre, University of Social Welfare and Rehabilitation Sciences, Tehran, Iran; 69 Faculty of Philosophy, Ss. Cyril and Methodius University in Skopje, Republic of North Macedonia; 70 Faculty of Philosophy, University of Ss. Cyril and Methodius in Trnava, Slovakia; 71 SAMRC Unit on Risk & Resilience in Mental Disorders, Department of Psychiatry & Neuroscience Institute, University of Cape Town, South Africa; 72 Austrian Public Health Institute, Vienna, Austria; 73 Département de Psychologie, Université du Québec à Trois-Rivières, Trois-Rivières, Canada; 74 South East Technological University, Waterford, Ireland; 75 Center for Excessive Gambling, Addiction Medicine, Lausanne University Hospital, Lausanne, Switzerland

**Keywords:** confirmatory factor analysis, impulsive behaviors, International Sex Survey, measurement invariance analysis, UPPS-P Impulsive Behavior Scale

## Abstract

The UPPS-P Impulsive Behavior Model and the various psychometric instruments developed and validated based on this model are well established in clinical and research settings. However, evidence regarding the psychometric validity, reliability, and equivalence across multiple countries of residence, languages, or gender identities, including gender-diverse individuals, is lacking to date. Using data from the International Sex Survey (*N* = 82,243), confirmatory factor analyses and measurement invariance analyses were performed on the preestablished five-factor structure of the 20-item short version of the UPPS-P Impulsive Behavior Scale to examine whether (a) psychometric validity and reliability and (b) psychometric equivalence hold across 34 country-of-residence-related, 22 language-related, and three gender-identity-related groups. The results of the present study extend the latter psychometric instrument’s well-established relevance to 26 countries, 13 languages, and three gender identities. Most notably, psychometric validity and reliability were evidenced across nine novel translations included in the present study (i.e., Croatian, English, German, Hebrew, Korean, Macedonian, Polish, Portuguese—Portugal, and Spanish—Latin American) and psychometric equivalence was evidenced across all three gender identities included in the present study (i.e., women, men, and gender-diverse individuals).

## Introduction

*Impulsivity* is a psychological construct included in most prominent personality models (Whiteside & Lynam, 2001) and is one of the most frequently encountered diagnostic criteria in nosography manuals (American Psychiatric Association, 2022; World Health Organization, 2019). Consistently, impulsivity is transdiagnostically implicated in the etiology of numerous psychopathological and neurological disorders (Berg et al., 2015; Evenden, 1999; Rochat et al., 2018). Among the most dominant impulsivity models, the UPPS-P Impulsive Behavior Model (Cyders et al., 2007; Whiteside & Lynam, 2001) conceptualizes impulsivity as a multidimensional construct encompassing five distinct facets, namely, (a) *lack of premeditation* (lack of reflection on the potential consequences of actions preceding their emission), (b) *positive urgency* (emission of sudden actions in intense positive emotional contexts), (c) *sensation seeking* (tendency to appreciate and seek excitement and to be open to new experiences), (d) *negative urgency* (emission of sudden actions in intense negative emotional contexts), and (e) *lack of perseverance* (difficulty focusing on the completion of demanding or monotonous tasks).

Initially, the UPPS-P Impulsive Behavior Model was developed based on four different impulsivity-related facets in the Revised NEO Personality Inventory (i.e., impulsiveness, excitement seeking, self-discipline, and deliberation) (Costa & McCrae, 2008) and 17 classic scales or subscales measuring impulsivity (Whiteside & Lynam, 2001). By federating 21 coexisting conceptualizations of impulsivity and thereby correcting the jingle (i.e., distinct constructs designated by one same label) and jangle (i.e., distinct labels designating one same construct) fallacies that characterized the research field of impulsivity, the UPPS-P Impulsive Behavior Model received great interest and exerted a significant impact on subsequent impulsivity research. Several psychometric instruments were developed and validated based on the UPPS-P Impulsive Behavior Model, such as the original 59-item UPPS-P Impulsive Behavior Scale (Cyders et al., 2007; Whiteside & Lynam, 2001), the 20-item short French version of the UPPS-P Impulsive Behavior Scale (Billieux et al., 2012), and the 20-item short English version of the UPPS-P Impulsive Behavior Scale (Cyders et al., 2014). Given their well-established psychometric properties and relevance for various problematic behaviors and mental disorders, UPPS-P Impulsive Behavior Scales were adapted to numerous languages (Bteich et al., 2017; Cándido et al., 2012; d’Orta et al., 2015; Wang et al., 2020; Zsila et al., 2020) and populations such as children (Geurten et al., 2021), adolescents (d’Acremont & Van der Linden, 2005), patients in psychiatric emergency settings (Dugré et al., 2019), patients with substance use disorders (Calzada et al., 2017; Kempeneers et al., 2023; Sánchez-Domínguez et al., 2022), and patients with neurological disorders (Rochat et al., 2008, 2010).

Recently, several studies examined and fully established the measurement invariance of UPPS-P Impulsive Behavior Scales across different groups, such as age-related (Argyriou et al., 2020; Donati et al., 2021; Wang et al., 2020), ethnicity-related (Liu et al., 2023; Stevens et al., 2018; Watts et al., 2020), gender-identity-related (Donati et al., 2021; Gialdi et al., 2021; Watts et al., 2020), and sex-related (Argyriou et al., 2020; Cyders, 2013) groups. Measurement invariance assesses whether the assumption of equivalence of a psychological construct—as measured by a corresponding psychometric instrument—holds across certain defined groups, which is a prerequisite for suggesting that the said psychological construct has comparable meaning for these groups (Jeong & Lee, 2019; Milfont & Fischer, 2010; Putnick & Bornstein, 2016). However, despite the extensiveness of UPPS-P-related research, no study published to date has examined the measurement invariance of UPPS-P Impulsive Behavior Scales across country-of-residence-related or language-related groups, and although three studies published to date have examined the measurement invariance of UPPS-P Impulsive Behavior Scales across gender-identity-related groups (Donati et al., 2021; Gialdi et al., 2021; Watts et al., 2020), none included gender-diverse individuals. Therefore, evidence is lacking to date regarding whether the impulsive behavior dimensions—as assessed by UPPS-P Impulsive Behavior Scales—have comparable meaning for individuals across the latter groups and whether one can engage in cross-group comparison analyses of the composite factor scores of UPPS-P Impulsive Behavior Scales. Moreover, in the interest of including country-of-residence-related, language-related, and gender-identity-related groups for which evidence is lacking to date, such evidence is critically warranted to support the integration of the assessment of impulsive behavior dimensions in clinical and research settings with respect to underrepresented and underserved groups.

In the present study, to address the abovementioned gaps, we probed the preestablished five-factor structure and the measurement invariance of the 20-item short version of the UPPS-P Impulsive Behavior Scale developed and validated by Billieux et al. (2012) across 34 country-of-residence-related, 22 language-related, and three gender-identity-related groups, with the overarching aims to examine whether (a) psychometric validity and reliability and (b) psychometric equivalence hold across different groups.

## Method

### Participants and Procedure

The total sample comprised participants recruited in the context of the International Sex Survey (Bőthe et al., 2021), a collaborative study conducted across 42 countries^1^, all of which received ethical clearance directly from local ethics committees or indirectly from the principal investigators’ institution’s local ethics committee (e.g., the Institutional Review Board of the Eötvös Loránd University, Budapest, Hungary). Detailed ethical information is available from the Open Science Framework (https://osf.io/e93kf). Participation consisted of completing online sociodemographic information questions and self-administered psychometric instruments, one of which, the 20-item short version of the UPPS-P Impulsive Behavior Scale (Billieux et al., 2012), was investigated in the present study.

The total sample included 82,243 participants from the general population of legal age residing across 42 countries and speaking 26 different languages. The age of the participants ranged between 18 and 99 (*M* = 32.391, *SD* = 12.524) years, with 56.995% identifying as women, 39.577% as men, and 3.384% as gender-diverse individuals. Detailed sociodemographic information is available from the Open Science Framework (https://osf.io/cj658).

### Materials

The 20-item short version of the UPPS-P Impulsive Behavior Scale (Billieux et al., 2012) is a self-administered psychometric instrument. This instrument assesses the applicability of 20 statements related to the five different dimensions of the UPPS-P Impulsive Behavior Model, namely, (a) *lack of premeditation* (e.g., Item 13: “I usually make up my mind through careful reasoning.”), (b) *positive urgency* (e.g., reverse-scored Item 15*: “I tend to act without thinking when I am really excited.”), (c) *sensation seeking* (e.g., reverse-scored Item 9*: “I quite enjoy taking risks.”), (d) *negative urgency* (e.g., reverse-scored Item 12*: “I often make matters worse because I act without thinking when I am upset.”), and (e) *lack of perseverance* (e.g., Item 8: “I finish what I start.”) (Cyders et al., 2007; Whiteside & Lynam, 2001). Each of the five instrument’s dimensions includes four items that are scored (or reverse-scored) on a 4-point Likert-type scale (from 1 = *strongly agree* to 4 = *strongly disagree*) and that provide composite factor scores likewise ranging from 1 (i.e., the lowest level of endorsement of the corresponding impulsive behavior dimension) to 4 (i.e., the highest level of endorsement of the corresponding impulsive behavior dimension).

In the context of the International Sex Survey, the 20-item short version of the UPPS-P Impulsive Behavior Scale (Billieux et al., 2012) was first adapted from French—its original language—to English based on prevalidated English items from the original 59-item UPPS-P Impulsive Behavior Scale (Cyders et al., 2007; Whiteside & Lynam, 2001), then adapted from English to all target languages (for which no corresponding validated translation was available) following a preestablished translation protocol (Beaton et al., 2000). Detailed information regarding the materials in all 26 languages included in the International Sex Survey is available from the Open Science Framework (https://osf.io/b5tdw).

### Data Analytic Plan

Data analysis was performed following a preregistered data analytic plan available from the Open Science Framework (https://doi.org/10.17605/OSF.IO/DK78R). All analyses were performed using *R* version 4.3.0 (R Core Team, 2023). Detailed information regarding the analyses and the code is available from the Open Science Framework (https://doi.org/10.17605/OSF.IO/UVPC2; https://doi.org/10.17605/OSF.IO/AS8R5). The corresponding data are not available from the Open Science Framework as the International Sex Survey involves sensitive data.

Item-level and construct-level missingness due to partial response rate (1.160% of the participants responded to between one and all but one item) on the 20-item short version of the UPPS-P Impulsive Behavior Scale was handled through multiple imputations (i.e., five iterations of five imputations of predictive mean matching) (Newman, 2014) using the *R* package *mice* version 3.16.0 (van Buuren et al., 2023).

Confirmatory factor analyses and measurement invariance analyses of the 20-item short version of the UPPS-P Impulsive Behavior Scale were performed within the framework of structural equation modeling analysis using the *R* packages *lavaan* version 0.6-15 (Rosseel et al., 2023) and *semTools* version 0.5-6 (Jorgensen et al., 2022).

Confirmatory factor analyses of the 20-item short version of the UPPS-P Impulsive Behavior Scale were performed with respect to its preestablished five-factor structure on the total sample (*N* = 82,243) and on all country-of-residence-related, language-related, and gender-identity-related groups that presented sufficient subsample size (*n*≥460) according to Monte Carlo simulation analyses (Type I error probability α = 0.050; Type II error probability β = 0.800) (Muthén & Muthén, 2002) conducted in the context of the preregistered data analytic plan. To fit the structural equation models, weighted least squares mean-and-variance-adjusted robust estimation methods were employed (Finney & di Stefano, 2013). To assess the quality of the structural equation models’ adjustment to the data, three conventional model fit indices were employed: the comparative fit index (CFI), the Tucker–Lewis index (TLI), and the root mean square error of approximation (RMSEA) along with its corresponding 90% confidence interval (Kline & Little, 2023). Following the preregistered data analytic plan, good fit was determined by a CFI ≥ 0.950, a TLI ≥ 0.950, and an RMSEA ≤ 0.050, while acceptable fit was determined by a CFI ≥ 0.900, a TLI ≥ 0.900, and an RMSEA ≤ 0.080 (Browne & Cudeck, 1992; F. Chen et al., 2008; Kenny et al., 2015; Marsh et al., 2005; Schermelleh-Engel et al., 2003).

Measurement invariance analyses of the 20-item short version of the UPPS-P Impulsive Behavior Scale were performed with respect to its preestablished five-factor structure on all country-of-residence-related, language-related, and gender-identity-related groups for which the abovementioned procedure yielded confirmatory factor analysis models with acceptable fit. Four measurement invariance hypotheses were sequentially and hierarchically examined by incrementally imposing cross-group equality constraints on the initial unconstrained models’ parameters (i.e., the preestablished five-factor structure of the 20-item short version of the UPPS-P Impulsive Behavior Scale) (Kline & Little, 2023). In the first examined measurement invariance hypothesis, item thresholds’ invariance, cross-group equality constraints were imposed on the model-implied non-null τ unstandardized estimates^2^ (Wu & Estabrook, 2016). Item thresholds refer to the boundaries between adjacent categories in ordered observed variables by relating the latter boundaries to points on a continuous latent normal distribution (Kline & Little, 2023). Item thresholds’ invariance implies that the item thresholds with respect to the 20-item short version of the UPPS-P Impulsive Behavior Scale are equivalent across groups and, therefore, that individuals across different groups interpret the 4-point Likert-type scales of the impulsive behavior items similarly (Jeong & Lee, 2019; Milfont & Fischer, 2010; Putnick & Bornstein, 2016). In the second examined measurement invariance hypothesis, factor loadings’ invariance, cross-group equality constraints were imposed on the model-implied non-null λ unstandardized estimates (Wu & Estabrook, 2016). Factor loadings refer to the magnitudes of associations between latent variables and observed variables (Kline & Little, 2023). Factor loadings’ invariance implies that the factor loadings with respect to the 20-item short version of the UPPS-P Impulsive Behavior Scale are equivalent across groups and, therefore, that the magnitudes of associations between impulsive behavior dimensions and their corresponding items are similar for individuals across different groups (Jeong & Lee, 2019; Milfont & Fischer, 2010; Putnick & Bornstein, 2016). In the third examined measurement invariance hypothesis, item intercepts’ invariance, cross-group equality constraints were imposed on the model-implied non-null ν unstandardized estimates (Wu & Estabrook, 2016). Item intercepts refer to the observed variables’ means considering that all latent variables equal zero (Kline & Little, 2023). Item intercepts’ invariance implies that the item intercepts with respect to the 20-item short version of the UPPS-P Impulsive Behavior Scale are equivalent across groups and, therefore, that individuals across different groups who present similar levels of endorsement of the impulsive behavior dimensions also present similar levels of endorsement of their corresponding items (Jeong & Lee, 2019; Milfont & Fischer, 2010; Putnick & Bornstein, 2016). Of note, accepting all three aforementioned measurement invariance hypotheses would imply that the impulsive behavior dimensions—as assessed by the 20-item short version of the UPPS-P Impulsive Behavior Scale—have comparable meaning for individuals across different groups and that one can engage in cross-group comparison analyses of the composite factor scores of the latter psychometric instrument (Jeong & Lee, 2019; Milfont & Fischer, 2010; Putnick & Bornstein, 2016). In the fourth and last examined measurement invariance hypothesis, item residuals’ invariance, cross-group equality constraints were imposed on the model-implied non-null θ unstandardized estimates (Wu & Estabrook, 2016). Item residuals refer to the observed variables’ sum of unique and error variances (Kline & Little, 2023). Item intercepts’ invariance implies that the item residuals with respect to the 20-item short version of the UPPS-P Impulsive Behavior Scale are equivalent across groups and, therefore, that the measurement error between impulsive behavior dimensions and their corresponding items are similar for individuals across different groups (Jeong & Lee, 2019; Milfont & Fischer, 2010; Putnick & Bornstein, 2016). Following the preregistered data analytic plan, acceptable measurement invariance between sequential structural equation models’ fit was determined by a Δ_CFI_≥–0.010, a Δ_TLI_≥–0.010, and a Δ_RMSEA_≤ 0.015 (F. F. Chen, 2007; Cheung & Rensvold, 2002). If the latter decision rules were not met, cross-group equality constraints to the corresponding sequential structural equation models’ model-implied non-null unstandardized estimates were released based on univariate chi-square tests’ statistics (i.e., “modification indices”) until partial measurement invariance (Milfont & Fischer, 2010) was supported by the data (likewise determined by a Δ_CFI_≥–0.010, a Δ_TLI_≥–0.010, and a Δ_RMSEA_≤ 0.015).

Cross-group comparison analyses of the composite factor scores of the 20-item short version of the UPPS-P Impulsive Behavior Scale were performed with respect to its preestablished five-factor structure on all country-of-residence-related, language-related, and gender-identity-related groups for which measurement invariance analyses supported item intercepts’ invariance (i.e., “strong invariance”). Cross-group comparison analyses were performed using two-sided Kruskal–Wallis rank sum tests, and pairwise cross-group comparison analyses were performed using one-sided Wilcoxon rank sum tests. Cross-group comparison analysis results were interpreted in light of their probability values and effect sizes: negligible effect size was determined by an *r* < 0.100 or an η^2^ < 0.010, small effect size was determined by an *r*≥0.100 or an η^2^≥0.010, moderate effect size was determined by an *r*≥0.250 or an η^2^≥0.0625, and large effect size was determined by an *r*≥0.500 or an η^2^≥0.250 (Cohen, 1992, 2013).

## Results

Factor-level and item-level descriptive analyses of the 20-item short version of the UPPS-P Impulsive Behavior Scale derived from analyses performed on the total sample (*N* = 82,243) yielded the descriptive values shown in Table 1.

**Table 1:** Factor-Level and Item-Level Descriptive Analyses of the 20-Item Short Version of the UPPS-P Impulsive Behavior Scale (Billieux et al., 2012) Derived From Analyses Performed on the Total Sample (*N* = 82,243).

Factor	*M*	*SD*	γ_1_	γ_2_	α	ω	Item	*M*	*SD*	γ_1_	γ_2_
Lack of premeditation	1.800	0.548	0.443	0.164	0.794	0.795	1. I usually think carefully before doing anything.	1.785	0.693	0.613	0.314
							6. My thinking is usually careful and purposeful.	1.834	0.727	0.589	0.110
							13. I usually make up my mind through careful reasoning.	1.818	0.687	0.532	0.226
							19. Before making up my mind, I consider all the advantages and disadvantages.	1.763	0.674	0.566	0.231
Positive urgency	2.368	0.642	0.044	–0.288	0.722	0.736	2*. When I am really excited, I tend not to think on the consequences of my actions.	2.304	0.875	0.078	−0.763
							10*. When overjoyed, I feel like I can’t stop myself from going overboard.	2.255	0.898	0.196	−0.771
							15*. I tend to act without thinking when I am really excited.	2.189	0.844	0.202	−0.657
							20*. When I am very happy, I feel like it is OK to give in to cravings or overindulge.	2.725	0.823	−0.406	−0.271
Sensation seeking	2.424	0.672	–0.008	–0.405	0.771	0.789	3*. I sometimes like doing things that are a bit frightening.	2.262	0.941	0.039	−1.060
							9*. I quite enjoy taking risks.	2.251	0.860	0.153	−0.697
							14*. I generally seek new and exciting experiences and activities.	2.592	0.827	−0.116	−0.526
							18*. I welcome new and exciting experiences and sensations, even if they are a little frightening and unconventional.	2.590	0.853	−0.258	−0.545
Negative urgency	2.266	0.700	0.157	–0.492	0.783	0.790	4*. When I am upset, I often act without thinking.	2.238	0.897	0.157	−0.834
							7*. In the heat of an argument, I will often say things that I later regret.	2.515	0.892	−0.070	−0.741
							12*. I often make matters worse because I act without thinking when I am upset.	2.089	0.886	0.365	−0.705
							17*. When I feel rejected, I will often say things that I later regret.	2.222	0.907	0.179	−0.859
Lack of perseverance	1.943	0.624	0.423	–0.068	0.822	0.841	5. I generally like to see things through to the end.	1.765	0.738	0.721	0.179
							8. I finish what I start.	1.966	0.757	0.444	−0.161
							11. Once I start a project, I almost always finish it.	2.010	0.798	0.447	−0.291
							16. I am a productive person who always gets the job done.	2.032	0.788	0.465	−0.151

*Note. M* = variable’s mean; *SD* = variable’s standard deviation; γ_1_ = variable’s skew index; γ_2_ = variable’s kurtosis index; α = variable’s (confirmatory-factor-analysis-model-implied) Cronbach’s alpha internal consistency value; ω = variable’s (confirmatory-factor-analysis-model-implied) McDonald’s omega internal consistency value. Single asterisks indicate reverse-scored items. All reported values were obtained after handling multivariate missing data through multiple imputations (i.e., five iterations of five imputations of predictive mean matching) (Newman, 2014).

**Figure 1.**
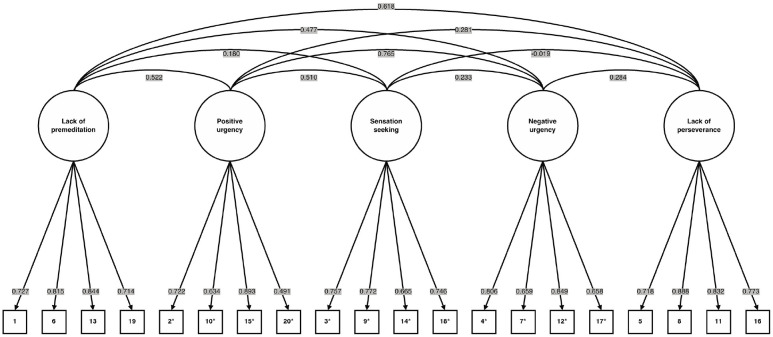
Confirmatory Factor Analysis Model of the 20-Item Short Version of the UPPS-P Impulsive Behavior Scale (Billieux et al., 2012) Derived From Analyses Performed on the Total Sample (*N* = 82,243). *Note.* Circles denote latent variables (i.e., factors). Squares denote observed variables (i.e., items). Arrows connecting latent variables to observed variables denote model-implied non-null λ standardized estimates (i.e., factor loadings). Lines connecting latent variables denote model-implied non-null φ standardized estimates (i.e., factor covariances). Single asterisks indicate reverse-scored items. All reported values were obtained after handling multivariate missing data through multiple imputations (i.e., five iterations of five imputations of predictive mean matching) (Newman, 2014).

Confirmatory factor analyses of the 20-item short version of the UPPS-P Impulsive Behavior Scale performed with respect to its preestablished five-factor structure on the total sample (*N* = 82,243) yielded the model-implied non-null λ and φ standardized estimates shown in Figure 1. The model-implied fit indices showed an acceptable to good quality of adjustment to the total sample, *N* = 82,243, χ^2^(160) = 52,867.560, *p* < 0.001, CFI = 0.957, TLI = 0.949, RMSEA [90% CI] = 0.063 [0.063, 0.064]. The model-implied Cronbach’s alpha and McDonald’s omega internal consistency values are shown in Table 1.

Confirmatory factor analyses of the 20-item short version of the UPPS-P Impulsive Behavior Scale performed with respect to its preestablished five-factor structure on all 34 country-of-residence-related, 22 language-related, and three gender-identity-related groups that presented sufficient subsample size according to the Monte Carlo simulation analyses conducted in the context of the preregistered data analytic plan (*n*≥460) yielded the model-implied fit indices, Cronbach’s alpha, and McDonald’s omega internal consistency values shown in Table 2. Of these, 26 country-of-residence-related, 13 language-related, and three gender-identity-related groups presented confirmatory factor analysis models with sufficient quality of adjustment to the data (see Table 2).

**Table 2:** Confirmatory Factor Analysis Models of the 20-Item Short Version of the UPPS-P Impulsive Behavior Scale (Billieux et al., 2012) Derived From Analyses Performed on the Country-of-Residence-Related, Language-Related, and Gender-Identity-Related Groups.

Groups	Group	*n*	χ^2^	*df*	*p*	CFI	TLI	RMSEA [90% CI]	α	ω
**Country-of-residence-related groups** (*N* = 42)	Algeria*	24	−	−	−	−	−	−	−	−
	**Australia**	639	701.397	160	<0.001	0.957	0.949	0.073 [0.067, 0.078]	[0.731, 0.838]	[0.752, 0.863]
	**Austria**	746	441.274	160	<0.001	0.982	0.978	0.049 [0.043, 0.054]	[0.655, 0.810]	[0.660, 0.877]
	Bangladesh*	373	−	−	−	−	−	−	−	−
	**Belgium**	644	724.688	160	<0.001	0.944	0.934	0.074 [0.069, 0.080]	[0.620, 0.807]	[0.649, 0.826]
	Bolivia*	385	−	−	−	−	−	−	−	−
	Brazil**	3,579	4,225.728	160	<0.001	0.934	0.921	0.084 [0.082, 0.086]	[0.687, 0.826]	[0.707, 0.856]
	**Canada**	2,541	1,704.313	160	<0.001	0.971	0.966	0.062 [0.059, 0.064]	[0.701, 0.842]	[0.747, 0.869]
	**Chile**	1,173	980.706	160	<0.001	0.948	0.938	0.066 [0.062, 0.070]	[0.690, 0.796]	[0.694, 0.858]
	China**	2,428	2,896.063	160	<0.001	0.932	0.920	0.084 [0.081, 0.087]	[0.765, 0.802]	[0.773, 0.842]
	**Colombia**	1,913	1,469.391	160	<0.001	0.943	0.932	0.065 [0.062, 0.069]	[0.704, 0.788]	[0.714, 0.812]
	**Croatia**	2,390	2,206.876	160	<0.001	0.949	0.939	0.073 [0.070, 0.076]	[0.685, 0.853]	[0.708, 0.870]
	Czech Republic**	1,640	2,012.753	160	<0.001	0.929	0.916	0.084 [0.081, 0.087]	[0.667, 0.864]	[0.671, 0.870]
	Ecuador*	276	−	−	−	−	−	−		
	**France**	1,706	1,469.209	160	<0.001	0.964	0.957	0.069 [0.066, 0.073]	[0.738, 0.872]	[0.757, 0.879]
	**Germany**	3,271	1,651.953	160	<0.001	0.973	0.968	0.053 [0.051, 0.056]	[0.634, 0.833]	[0.647, 0.858]
	Gibraltar*	64	−	−	−	−	−	−		
	**Hungary**	11,200	10,356.581	160	<0.001	0.947	0.938	0.075 [0.074, 0.077]	[0.724, 0.832]	[0.744, 0.844]
	India*	194	−	−	−	−	−	−		
	Iraq*	99	−	−	−	−	−	−		
	**Ireland**	1,702	1,411.981	160	<0.001	0.956	0.948	0.068 [0.065, 0.071]	[0.734, 0.832]	[0.763, 0.850]
	**Israel**	1,334	1,075.832	160	<0.001	0.962	0.955	0.066 [0.062, 0.069]	[0.716, 0.828]	[0.727, 0.862]
	**Italy**	2,401	1,709.934	160	<0.001	0.966	0.960	0.064 [0.061, 0.066]	[0.757, 0.842]	[0.779, 0.858]
	Japan**	562	889.717	160	<0.001	0.924	0.910	0.090 [0.084, 0.096]	[0.669, 0.837]	[0.737, 0.836]
	Lithuania**	2,015	3,730.784	160	<0.001	0.890	0.869	0.105 [0.102, 0.108]	[0.679, 0.802]	[0.703, 0.993]
	**Malaysia**	1,170	1,065.731	160	<0.001	0.942	0.932	0.070 [0.066, 0.074]	[0.722, 0.789]	[0.736, 0.811]
	**Mexico**	2,137	1,903.226	160	<0.001	0.944	0.934	0.071 [0.069, 0.074]	[0.750, 0.789]	[0.762, 0.887]
	**New Zealand**	2,834	2,043.619	160	<0.001	0.966	0.959	0.064 [0.062, 0.067]	[0.739, 0.847]	[0.776, 0.873]
	**North Macedonia**	1,251	1,312.495	160	<0.001	0.926	0.912	0.076 [0.072, 0.080]	[0.685, 0.835]	[0.699, 0.861]
	Other***	1,177	−	−	−	−	−	−	−	−
	Panama*	333	−	−	−	−	−	−	−	−
	**Peru**	2,672	2,240.777	160	<0.001	0.941	0.930	0.070 [0.067, 0.072]	[0.710, 0.795]	[0.718, 0.844]
	**Poland**	9,892	4,815.148	160	<0.001	0.971	0.965	0.054 [0.053, 0.056]	[0.711, 0.828]	[0.713, 0.835]
	**Portugal**	2,262	2,065.304	160	<0.001	0.947	0.938	0.073 [0.070, 0.075]	[0.724, 0.834]	[0.750, 0.853]
	Slovakia**	1,134	2,329.792	160	<0.001	0.905	0.888	0.109 [0.105, 0.113]	[0.724, 0.821]	[0.737, 0.994]
	**South Africa**	1,849	1,628.273	160	<0.001	0.954	0.945	0.070 [0.067, 0.074]	[0.751, 0.832]	[0.767, 0.856]
	**South Korea**	1,464	1,458.545	160	<0.001	0.958	0.950	0.074 [0.071, 0.078]	[0.706, 0.873]	[0.738, 0.879]
	**Spain**	2,327	2,102.654	160	<0.001	0.941	0.929	0.072 [0.070, 0.075]	[0.736, 0.797]	[0.745, 0.878]
	**Switzerland**	1,144	953.921	160	<0.001	0.968	0.962	0.066 [0.062, 0.070]	[0.718, 0.867]	[0.727, 0.893]
	Taiwan**	2,668	4,482.619	160	<0.001	0.912	0.895	0.101 [0.098, 0.103]	[0.708, 0.802]	[0.720, 0.880]
	Turkey**	820	1,193.311	160	<0.001	0.917	0.901	0.089 [0.084, 0.094]	[0.631, 0.782]	[0.673, 0.842]
	**United Kingdom**	1,412	1,091.252	160	<0.001	0.962	0.954	0.064 [0.061, 0.068]	[0.741, 0.842]	[0.784, 0.858]
	**United States of America**	2,398	2,195.428	160	<0.001	0.956	0.947	0.073 [0.070, 0.076]	[0.748, 0.838]	[0.781, 0.864]
**Language-related groups** (*N* = 26)	Arabic*	142	−	−	−	−	−	−	−	−
	Bangla*	332	−	−	−	−	−	−	−	−
	**Croatian**	2,522	2,369.870	160	<0.001	0.947	0.937	0.074 [0.071, 0.077]	[0.687, 0.852]	[0.709, 0.869]
	Czech**	1,583	1,996.098	160	<0.001	0.926	0.912	0.085 [0.082, 0.089]	[0.663, 0.863]	[0.670, 0.868]
	Dutch**	518	806.930	160	<0.001	0.918	0.903	0.088 [0.082, 0.095]	[0.606, 0.809]	[0.630, 0.829]
	**English**	13,994	10,502.916	160	<0.001	0.959	0.951	0.068 [0.067, 0.069]	[0.742, 0.833]	[0.771, 0.858]
	**French**	3,941	2,841.435	160	<0.001	0.970	0.964	0.065 [0.063, 0.067]	[0.730, 0.870]	[0.757, 0.883]
	**German**	3,494	1,665.247	160	<0.001	0.978	0.973	0.052 [0.050, 0.054]	[0.626, 0.845]	[0.647, 0.868]
	**Hebrew**	1,315	1,062.185	160	<0.001	0.961	0.954	0.066 [0.062, 0.069]	[0.711, 0.825]	[0.722, 0.862]
	Hindi*	17	−	−	−	−	−	−	−	−
	**Hungarian**	10,937	10,362.625	160	<0.001	0.949	0.939	0.076 [0.075, 0.078]	[0.725, 0.822]	[0.745, 0.844]
	**Italian**	2,437	1,819.039	160	<0.001	0.965	0.959	0.065 [0.063, 0.068]	[0.755, 0.845]	[0.777, 0.860]
	Japanese**	466	828.093	160	<0.001	0.917	0.902	0.095 [0.088, 0.101]	[0.649, 0.832]	[0.740, 0.833]
	**Korean**	1,437	1,428.639	160	<0.001	0.959	0.951	0.074 [0.071, 0.078]	[0.706, 0.875]	[0.739, 0.880]
	Lithuanian**	2,094	3,880.183	160	<0.001	0.889	0.868	0.105 [0.103, 0.108]	[0.678, 0.802]	[0.704, 0.995]
	**Macedonian**	1,301	1,371.888	160	<0.001	0.926	0.912	0.076 [0.073, 0.080]	[0.688, 0.838]	[0.700, 0.865]
	Mandarin—Simplified**	2,474	2,890.516	160	<0.001	0.934	0.921	0.083 [0.080, 0.086]	[0.764, 0.804]	[0.772, 0.843]
	Mandarin—Traditional**	2,685	4,492.797	160	<0.001	0.912	0.895	0.100 [0.098, 0.103]	[0.708, 0.803]	[0.721, 0.879]
	**Polish**	10,343	5,057.192	160	<0.001	0.970	0.965	0.054 [0.053, 0.056]	[0.712, 0.830]	[0.713, 0.836]
	Portuguese—Brazil**	3,650	4,323.815	160	<0.001	0.934	0.922	0.084 [0.082, 0.087]	[0.685, 0.825]	[0.704, 0.857]
	**Portuguese—Portugal**	2,277	2,077.951	160	<0.001	0.946	0.936	0.073 [0.070, 0.075]	[0.729, 0.832]	[0.753, 0.853]
	Romanian*	75	−	−	−	−	−	−	−	−
	Slovak**	2,118	4,546.401	160	<0.001	0.902	0.884	0.114 [0.111, 0.117]	[0.716, 0.826]	[0.733, 1.020]
	**Spanish—Latin American**	8,926	6,962.316	160	<0.001	0.942	0.931	0.069 [0.068, 0.070]	[0.719, 0.794]	[0.728, 0.854]
	**Spanish—Spain**	2,312	2,086.469	160	<0.001	0.940	0.929	0.072 [0.069, 0.075]	[0.734, 0.794]	[0.743, 0.880]
	Turkish**	853	1,230.827	160	<0.001	0.921	0.907	0.089 [0.084, 0.093]	[0.627, 0.785]	[0.677, 0.847]
**Gender-identity-related groups** (*N* = 3)	**Gender-diverse individuals**	2,783	1,838.463	160	<0.001	0.960	0.953	0.061 [0.059, 0.064]	[0.735, 0.809]	[0.759, 0.831]
	**Men**	32,549	24,524.505	160	<0.001	0.951	0.942	0.068 [0.068, 0.069]	[0.719, 0.820]	[0.732, 0.840]
	**Women**	46,874	26,008.323	160	<0.001	0.962	0.955	0.059 [0.058, 0.059]	[0.724, 0.823]	[0.736, 0.840]

*Note. N* = number of groups; *n* = variable’s subsample size; χ^2^ = model’s chi-square; *df* = model’s chi-square’s degrees of freedom; *p* = model’s chi-square’s probability value; CFI = model’s comparative fit index; TLI = model’s Tucker–Lewis fit index; RMSEA [90% CI] = model’s root mean square error of approximation along with its corresponding 90% confidence interval; α = group’s (confirmatory-factor-analysis-model-implied) Cronbach’s alpha internal consistency values range; ω = group’s (confirmatory-factor-analysis-model-implied) McDonald’s omega internal consistency values range. Boldfaced groups indicate acceptable confirmatory factor analysis models supported by the data. Single asterisks indicate groups that presented insufficient subsample size (*n* < 460) according to Monte Carlo simulation analyses (Type I error probability α = 0.050; Type II error probability β = 0.800) conducted in the context of the preregistered data analytic plan (https://doi.org/10.17605/OSF.IO/DK78R). Double asterisks indicate groups that presented confirmatory factor analysis models with nonacceptable fit determined by a CFI < 0.900, a TLI < 0.900, or an RMSEA > 0.080 following the preregistered data analytic plan (https://doi.org/10.17605/OSF.IO/DK78R). Triple asterisks indicate country-of-residence-related groups that were not included as collaborating countries and country-of-residence-related groups that were included as collaborating countries at the time of publication of the International Sex Survey’s study protocol (Bőthe et al., 2021) but that did not receive timely ethical clearance from local ethics committees (i.e., Egypt, Iran, Pakistan, and Romania). All reported values were obtained after handling multivariate missing data through multiple imputations (i.e., five iterations of five imputations of predictive mean matching) (Newman, 2014).

Measurement invariance analyses of the 20-item short version of the UPPS-P Impulsive Behavior Scale performed with respect to its preestablished five-factor structure on all 26 country-of-residence-related, 13 language-related, and three gender-identity-related groups that presented sufficient subsample size according to the Monte Carlo simulation analyses conducted in the context of the preregistered data analytic plan (*n*≥460) and that presented confirmatory factor analysis models with sufficient quality of adjustment to the data yielded the model-implied fit indices shown in Table 3. For country-of-residence-related and language-related groups, factor loadings’ invariance (i.e., “weak invariance”) was supported by the data (see Table 3). Several cross-group equality constraints to the initial unconstrained models’ model-implied non-null ν and θ unstandardized estimates ought to be released for item intercepts’ partial invariance (i.e., “partial strong invariance”) and item residuals’ partial invariance (i.e., “partial strict invariance”) to be supported by the data (see Table 3). For gender-identity-related groups, item residuals’ invariance (i.e., “strict invariance”) was supported by the data (see Table 3).

**Table 3:** Measurement Invariance Analysis Models of the 20-Item Short Version of the UPPS-P Impulsive Behavior Scale (Billieux et al., 2012) Derived From Analyses Performed on the Country-of-Residence-Related, Language-Related, and Gender-Identity-Related Groups.

Groups	EC	χ^2^	*df*	*p*	CFI	TLI	RMSEA [90% CI]
**Country-of-residence-related groups** (*N* = 26)	**NA**	49,648.378	4,160	<0.001	0.958	0.950	0.066 [0.066, 0.067]
	**τ**	53,781.400	4,660	<0.001	0.955	0.952	0.065 [0.065, 0.066]
	**τ, λ**	56,687.719	5,035	<0.001	0.953	0.954	0.064 [0.064, 0.065]
	τ, λ, ν	85,277.051	5,410	<0.001	0.927	0.933	0.077 [0.077, 0.078]
	τ, λ, ν*	67,953.723	5,310	<0.001	0.943	0.947	0.069 [0.069, 0.069]
	τ, λ, ν, θ	96,115.556	5,910	<0.001	0.917	0.931	0.078 [0.078, 0.079]
	τ, λ, ν*, θ***	78,694.688	5,785	<0.001	0.933	0.943	0.071 [0.071, 0.072]
**Language-related groups** (*N* = 13)	**NA**	48,590.579	2,080	<0.001	0.958	0.951	0.067 [0.066, 0.067]
	**τ**	52,866.967	2,320	<0.001	0.955	0.952	0.066 [0.065, 0.066]
	**τ, λ**	56,272.131	2,500	<0.001	0.952	0.952	0.065 [0.065, 0.066]
	τ, λ, ν	87,499.038	2,680	<0.001	0.924	0.930	0.079 [0.079, 0.080]
	τ, λ, ν**	64,729.701	2,620	<0.001	0.944	0.948	0.069 [0.068, 0.069]
	τ, λ, ν, θ	99,900.935	2,920	<0.001	0.913	0.927	0.081 [0.081, 0.082]
	τ, λ, ν**, θ***	76,664.073	2,848	<0.001	0.934	0.943	0.072 [0.071, 0.072]
**Gender-identity-related groups** (*N* = 3)	**NA**	52,113.803	480	<0.001	0.958	0.950	0.063 [0.062, 0.063]
	**τ**	53,229.675	520	<0.001	0.957	0.953	0.061 [0.060, 0.061]
	**τ, λ**	50,658.153	550	<0.001	0.959	0.957	0.058 [0.057, 0.058]
	**τ, λ, ν**	52,489.692	580	<0.001	0.957	0.958	0.057 [0.057, 0.058]
	**τ, λ, ν, θ**	49,911.192	620	<0.001	0.960	0.963	0.054 [0.053, 0.054]

*Note. N* = number of groups; EC = cross-group equality constraints to the initial unconstrained models’ parameters (i.e., the preestablished five-factor structure of the 20-item short version of the UPPS-P Impulsive Behavior Scale) (Billieux et al., 2012); NA = no cross-group equality constraints to the initial unconstrained models’ model-implied non-null unstandardized estimates; τ = cross-group equality constraints to the initial unconstrained models’ model-implied non-null τ unstandardized estimates (i.e., item thresholds’ invariance); λ = cross-group equality constraints to the initial unconstrained models’ model-implied non-null λ unstandardized estimates (i.e., factor loadings’ invariance or “weak invariance”); ν = cross-group equality constraints to the initial unconstrained models’ model-implied non-null ν unstandardized estimates (i.e., item intercepts’ invariance or “strong invariance”); θ = cross-group equality constraints to the initial unconstrained models’ model-implied non-null θ unstandardized estimates (i.e., item residuals’ invariance or “strict invariance”); χ^2^ = model’s chi-square; *df* = model’s chi-square’s degrees of freedom; *p* = model’s chi-square’s probability value; CFI = model’s comparative fit index; TLI = model’s Tucker–Lewis fit index; RMSEA [90% CI] = model’s root mean square error of approximation along with its corresponding 90% confidence interval. Boldfaced cross-group equality constraints to the initial unconstrained models’ parameters indicate acceptable measurement invariance analysis models supported by the data. Single asterisks indicate that for Item 3* (sensation seeking), Item 14* (sensation seeking), Item 20* (positive urgency), and Item 10* (positive urgency), cross-group equality constraints to the initial unconstrained model’s model-implied non-null ν unstandardized estimates were released (i.e., partial item intercepts’ invariance or “partial strong invariance”). Double asterisks indicate that, for Item 3* (sensation seeking), Item 14* (sensation seeking), Item 20* (positive urgency), Item 10* (positive urgency), and Item 5 (lack of perseverance), cross-group equality constraints to the initial unconstrained model’s model-implied non-null θ unstandardized estimates were released (i.e., partial item intercepts’ invariance or “partial strong invariance”). Triple asterisks indicate that, for Item 5 (lack of perseverance), cross-group equality constraints to the initial unconstrained model’s model-implied non-null θ unstandardized estimates were released (i.e., partial item residuals’ invariance or “partial strict invariance”). All reported values were obtained after handling multivariate missing data through multiple imputations (i.e., five iterations of five imputations of predictive mean matching) (Newman, 2014).

Cross-group comparison analyses of the composite factor scores of the 20-item short version of the UPPS-P Impulsive Behavior Scale were performed with respect to its preestablished five-factor structure on groups for which item intercepts’ invariance (i.e., “strong invariance”) was supported by the data (i.e., all three gender-identity-related groups). Factor-level and descriptive analyses of the 20-item short version of the UPPS-P Impulsive Behavior Scale derived from analyses performed on all three gender-identity-related groups yielded the descriptive values shown in Table 4, two-sided Kruskal–Wallis rank sum tests yielded the results shown in Table 5, and one-sided Wilcoxon rank sum tests yielded the results shown in Table 6. All five two-sided Kruskal–Wallis rank sum tests were significant (*p* < 0.001); of these, four presented negligible effect sizes (η^2^∈ [0.004, 0.008]), whereas one presented a small effect size (η^2^ = 0.017) suggesting that the composite factor scores of negative urgency were not equal between women, men, and gender-diverse individuals (see Table 5). All 15 one-sided Wilcoxon rank sum tests were significant (*p* < 0.001; *p* < 0.010); of these, 13 presented negligible effect sizes (*r*∈ [0.011, 0.097]), whereas one presented a small effect size (*r* = 0.132) suggesting that the composite factor scores of negative urgency were higher for women than for men, and one presented a small effect size (*r* = 0.135) suggesting that the composite factor scores of lack of perseverance were lower for men than for gender-diverse individuals (see Table 6).

**Table 4:** Factor-Level Descriptive Analyses of the 20-Item Short Version of the UPPS-P Impulsive Behavior Scale (Billieux et al., 2012) Derived From Analyses Performed on all 3/3 Gender-Identity-Related Groups.

Groups	Factor	Group	*M*	*SD*	γ_1_	γ_2_
**Gender-identity-related groups** (*N* = 3)	**Lack of premeditation**	Gender-diverse individuals (*n* = 2,783)	1.901	0.594	0.383	−0.123
		Men (*n* = 32,549)	1.750	0.537	0.478	0.245
		Women (*n* = 46,874)	1.828	0.551	0.417	0.134
	**Positive urgency**	Gender-diverse individuals (*n* = 2,783)	2.551	0.670	−0.024	−0.377
		Men (*n* = 32,549)	2.334	0.637	0.041	−0.297
		Women (*n* = 46,874)	2.381	0.641	0.044	−0.280
	**Sensation seeking**	Gender-diverse individuals (*n* = 2,783)	2.600	0.704	−0.176	−0.439
		Men (*n* = 32,549)	2.478	0.656	−0.025	−0.346
		Women (*n* = 46,874)	2.376	0.676	0.019	−0.435
	**Negative urgency**	Gender-diverse individuals (*n* = 2,783)	2.379	0.750	0.107	−0.608
		Men (*n* = 32,549)	2.151	0.690	0.246	−0.477
		Women (*n* = 46,874)	2.339	0.694	0.100	−0.464
	**Lack of perseverance**	Gender-diverse individuals (*n* = 2,783)	2.234	0.680	0.115	−0.459
		Men (*n* = 32,549)	1.901	0.610	0.444	−0.016
		Women (*n* = 46,874)	1.955	0.625	0.415	−0.068

*Note. N* = number of groups; *n* = variable’s subsample size; *M* = variable’s mean; *SD* = variable’s standard deviation; γ_1_ = variable’s skew index; γ_2_ = variable’s kurtosis index. All reported values were obtained after handling multivariate missing data through multiple imputations (i.e., five iterations of five imputations of predictive mean matching) (Newman, 2014).

**Table 5:** Cross-Group Comparison Analyses of the 20-Item Short Version of the UPPS-P Impulsive Behavior Scale (Billieux et al., 2012) Derived From Analyses Performed on all 3/3 Gender-Identity-Related Groups.

Groups	Factor	*H*_0_	*H*_1_	*H*	*df*	*p*	η^2^
**Gender-identity-related groups** (*N* = 3)	**Lack of premeditation**	=	!=	465.866	2	<0.001	0.006
	**Positive urgency**	=	!=	301.272	2	<0.001	0.004
	**Sensation seeking**	=	!=	614.536	2	<0.001	0.007
	**Negative urgency**	=	!=	1437.843	2	<0.001	0.017
	**Lack of perseverance**	=	!=	696.251	2	<0.001	0.008

*Note. N* = number of groups; *H*_0_ = null hypothesis; *H*_1_ = alternative hypothesis; *H* = two-sided Kruskal–Wallis rank sum test’s statistic; *df* = two-sided Kruskal–Wallis rank sum test’s degrees of freedom; *p* = two-sided Kruskal–Wallis rank sum test’s probability value; η^2^ = two-sided Kruskal–Wallis rank sum test’s effect size. All reported values were obtained after handling multivariate missing data through multiple imputations (i.e., five iterations of five imputations of predictive mean matching) (Newman, 2014).

**Table 6:** Pairwise Cross-Group Comparison Analyses of the 20-Item Short Version of the UPPS-P Impulsive Behavior Scale (Billieux et al., 2012) Derived From Analyses Performed on all 3/3 Gender-Identity-Related Groups.

Groups	Factor	Group 1	*H*_0_	*H*_1_	Group 2	*W*	*df*	*p*	*r*
**Gender-identity-related groups** (*N* = 3)	**Lack of premeditation**	Gender-diverse individuals	=	>	Men	51,894,871.500	1	<0.001	0.069
		Gender-diverse individuals	=	>	Women	69,683,774.500	1	<0.001	0.028
		Men	=	<	Women	701,403,651.000	1	<0.001	0.070
	**Positive urgency**	Gender-diverse individuals	=	>	Men	53,561,957.000	1	<0.001	0.086
		Gender-diverse individuals	=	>	Women	74,625,177.000	1	<0.001	0.058
		Men	=	<	Women	732,515,650.500	1	<0.001	0.034
	**Sensation seeking**	Gender-diverse individuals	=	>	Men	50,225,536.500	1	<0.001	0.051
		Gender-diverse individuals	=	>	Women	77,405,400.500	1	<0.001	0.075
		Men	=	>	Women	827,658,206.000	1	<0.001	0.073
	**Negative urgency**	Gender-diverse individuals	=	>	Men	53,177,247.000	1	<0.001	0.082
		Gender-diverse individuals	=	>	Women	67,057,798.000	1	0.006	0.011
		Men	=	<	Women	645,474,284.000	1	<0.001	0.132
	**Lack of perseverance**	Gender-diverse individuals	=	>	Men	58,305,963.000	1	<0.001	0.135
		Gender-diverse individuals	=	>	Women	80,994,000.000	1	<0.001	0.097
		Men	=	<	Women	725,834,660.500	1	<0.001	0.042

*Note. N* = number of groups; *H*_0_ = null hypothesis; *H*_1_ = alternative hypothesis; *W* = one-sided Wilcoxon rank sum test’s statistic; *df* = one-sided Wilcoxon rank sum test’s degrees of freedom; *p* = one-sided Wilcoxon rank sum test’s probability value; *r* = one-sided Wilcoxon rank sum test’s effect size. All reported values were obtained after handling multivariate missing data through multiple imputations (i.e., five iterations of five imputations of predictive mean matching) (Newman, 2014).

## Discussion

The UPPS-P Impulsive Behavior Model (Cyders et al., 2007; Whiteside & Lynam, 2001) and the various psychometric instruments developed and validated based on this model are well-established in clinical and research settings. However, evidence regarding the psychometric validity, reliability, and equivalence across multiple countries of residence, languages, or gender identities, including gender-diverse individuals, had been lacking to date. In the present study, we addressed the aforementioned gaps by probing the preestablished five-factor structure and the measurement invariance of the 20-item short version of the UPPS-P Impulsive Behavior Scale (Billieux et al., 2012) across 34 country-of-residence-related, 22 language-related, and three gender-identity-related groups, with the overarching aims to examine whether (a) psychometric validity and reliability and (b) psychometric equivalence held across different groups.

First, our confirmatory factor analysis results showed that the preestablished five-factor structure of the 20-item short version of the UPPS-P Impulsive Behavior Scale yielded an acceptable to good quality of adjustment to the data across the total sample, 26 country-of-residence-related groups, 13 language-related groups, and three gender-identity-related groups. In line with and in addition to prior UPPS-P-related literature evaluating the psychometric properties of UPPS-P Impulsive Behavior Scales, our confirmatory factor analysis results suggest that the psychological construct of impulsivity—as assessed by the 20-item short version of the UPPS-P Impulsive Behavior Scale—is valid and reliable across an extended number of countries, languages, and gender identities.

With respect to the translations that were adapted from the 20-item short version of the UPPS-P Impulsive Behavior Scale (Billieux et al., 2012), validated in prior UPPS-P-related literature, and employed in our study, our confirmatory factor analysis results regarding the French (Billieux et al., 2012), Hungarian (Zsila et al., 2020), Italian (d’Orta et al., 2015), and Spanish–Spain (Cándido et al., 2012) validated translations supported and reinforced their preestablished psychometric validity and reliability. The sole exception concerns the validated Mandarin–Simplified (Wang et al., 2020) translation. Notably, it was previously highlighted that an item included in the dimension of lack of perseverance (i.e., Item 11: “Once I start a project, I almost always finish it.”) might not tap its corresponding psychological construct due to psycholinguistic factors, which are likely to account for this nuance with respect to the Mandarin–Simplified validated translation (Wang et al., 2020).

With respect to the novel translations that were adapted from the 20-item short version of the UPPS-P Impulsive Behavior Scale (Billieux et al., 2012) following a preestablished translation protocol (Beaton et al., 2000) and employed in our study, our confirmatory factor analysis results regarding the Croatian, English, German, Hebrew, Korean, Macedonian, Polish, Portuguese–Portugal, and Spanish–Latin American novel translations supported their psychometric validity and reliability. In light of these promising results, we, therefore, invite researchers to engage in further psychometric validation of the aforementioned novel translations of the 20-item short version of the UPPS-P Impulsive Behavior Scale available from the Open Science Framework (https://osf.io/b5tdw).

Second, our measurement invariance analysis results showed that, whereas measurement invariance of the preestablished five-factor structure of the 20-item short version of the UPPS-P Impulsive Behavior Scale partially held across country-of-residence-related and language-related groups, it fully held across gender-identity-related groups.

With respect to country-of-residence-related and language-related groups, our measurement invariance analysis results showed that (a) the preestablished five-factor structure of the 20-item short version of the UPPS-P Impulsive Behavior Scale, (b) the 4-point Likert-type scales of its impulsive behavior items, and (c) the magnitudes of associations between its impulsive behavior dimensions and their corresponding items were fully invariant for individuals across such groups, therefore fully establishing “weak invariance” (Jeong & Lee, 2019; Milfont & Fischer, 2010; Putnick & Bornstein, 2016). Our results also showed that (d) the levels of endorsement of its impulsive behavior items (considering similar levels of endorsement of their corresponding dimensions) and (e) the measurement error between impulsive behavior dimensions and their corresponding items were partially invariant for individuals across such groups, therefore partially establishing “strict invariance” (Jeong & Lee, 2019; Milfont & Fischer, 2010; Putnick & Bornstein, 2016). Item intercepts’ partial invariance (i.e., “partial strong invariance”) analysis results provided insight into which impulsive behavior items reflected differential item functioning (DIF). The DIF denotes instances where individuals across different groups who present similar levels of endorsement of a dimension present dissimilar probabilities of responding similarly to some or all of the said dimension’s corresponding items (Kline & Little, 2023). With respect to country-of-residence-related and language-related groups, our results highlighted five instances of DIF: two relating to the dimension of positive urgency (i.e., Item 10*: “When overjoyed, I feel like I can’t stop myself from going overboard.” and Item 20*: “When I am very happy, I feel like it is OK to give in to cravings or overindulge.”), two relating to the dimension of sensation seeking (i.e., Item 3*: “I sometimes like doing things that are a bit frightening.” and Item 14*: “I generally seek new and exciting experiences and activities.”), and one relating to the dimension of lack of perseverance (i.e., Item 5: “I generally like to see things through to the end.”). Discussing the interpretation of partial measurement invariance results is a critical notion that ought to be addressed by researchers performing measurement invariance analyses. If erroneous, suggesting that a psychological construct has comparable meaning across certain groups may yield potentially significant implications, such as biased and invalid cross-group comparisons (Jeong & Lee, 2019). In light of our measurement invariance results across country-of-residence-related and language-related groups, as (a) cross-group equality constraints to the initial unconstrained models’ parameters ought to be released for a fourth of all items for “partial strict invariance” over “weak invariance” to be supported by the data, and as (b) there is no prior UPPS-P-related literature to support theoretical justification regarding the comparability of the meaning of the impulsive behavior dimensions for individuals across such groups, we recommend adopting a conservative approach and not interpreting that the psychological construct of impulsivity—as assessed by the 20-item short version of the UPPS-P Impulsive Behavior Scale—has comparable meaning across country-of-residence-related and language-related groups. In line with our aforementioned recommendation, we, therefore, did not engage in cross-group comparison analyses of the composite factor scores of the 20-item short version of the UPPS-P Impulsive Behavior Scale with respect to the country-of-residence-related and language-related groups.

Considering the intersections between country-of-residence-related and language-related groups, it is tenable that the latter might contribute to a certain degree of convergence in our confirmatory factor analysis and measurement invariance analysis results across such groups. With respect to the psychometric validity and reliability of the 20-item short version of the UPPS-P Impulsive Behavior Scale, our confirmatory factor analysis results across country-of-residence-related groups examined in the present study reflect those of their respective majority-language-related group. For instance, the proportion of the majority-language-related group French by the country-of-residence-related group Switzerland equaled 83.042%, and the psychometric validity and reliability of the 20-item short version of the UPPS-P Impulsive Behavior Scale were supported for both latter groups. For another instance, the proportion of the majority-language-related group Czech by the country-of-residence-related group Czech Republic equaled 96.037%, and the psychometric validity and reliability of the 20-item short version of the UPPS-P Impulsive Behavior Scale were not supported for both latter groups. Although the proportion of the majority-language-related group Dutch by the country-of-residence-related group Belgium equaled 73.137%, this constitutes the sole exception among all country-of-residence-related groups examined in the present study, as the psychometric validity and reliability of the 20-item short version of the UPPS-P Impulsive Behavior Scale were supported for the latter country-of-residence-related group but not for the latter language-related group. In this perspective, our confirmatory factor analysis and measurement invariance analysis results with respect to country-of-residence-related groups are liable to be partially explained by their intersections with language-related groups and, to a lesser extent, *vice versa*. Detailed information regarding the intersections of country-of-residence-related and language-related groups is available from the Open Science Framework (https://doi.org/10.17605/OSF.IO/TDEJW).

With respect to gender-identity-related groups, our measurement invariance analysis results showed that (a) the preestablished five-factor structure of the 20-item short version of the UPPS-P Impulsive Behavior Scale, (b) the 4-point Likert-type scales of its impulsive behavior items, (c) the magnitudes of associations between its impulsive behavior dimensions and their corresponding items, (d) the levels of endorsement of its impulsive behavior items (considering similar levels of endorsement of their corresponding dimensions) and (e) the measurement error between impulsive behavior dimensions and their corresponding items were fully invariant for individuals across such groups. Therefore, the impulsive behavior dimensions—as assessed by the 20-item short version of the UPPS-P Impulsive Behavior Scale—have comparable meaning for individuals across the aforementioned groups (Jeong & Lee, 2019; Milfont & Fischer, 2010; Putnick & Bornstein, 2016). Our results extend those of the three studies published to date which examined and established the measurement invariance of UPPS-P Impulsive Behavior Scales across gender-identity-related groups (Donati et al., 2021; Gialdi et al., 2021; Watts et al., 2020) to gender-diverse individuals. In addition, our cross-group comparison analysis results suggested that with respect to the impulsive behavior dimensions, differences between women, men, and gender-diverse individuals were mostly negligible or small.

Our study contains limitations that ought to be acknowledged. First, the psychometric properties of the 20-item short version of the UPPS-P Impulsive Behavior Scale were evaluated by gathering construct validity and internal consistency reliability evidence. In contrast, other types of evidence (e.g., convergent validity, test–retest reliability) were not gathered. However, we do not consider this a critical limitation, as the psychometric properties of the latter psychometric instrument are well established. Second, measurement invariance analyses of the 20-item short version of the UPPS-P Impulsive Behavior Scale were performed within the framework of multiple-group confirmatory factor analysis (MGCFA), which imposes strict cross-group equality constraints and, by extension, constitutes a conservative approach to psychometric equivalence. Measurement invariance analyses can be performed within other frameworks, such as the alignment method, which does not impose strict cross-group equality constraints and, by extension, constitutes a liberal approach to psychometric equivalence (Asparouhov & Muthén, 2014). In this perspective, the framework in which measurement invariance analyses were performed might have contributed to their corresponding results. However, the alignment approach is particularly recommended when examining large numbers of groups or parameters as the computational complexity of the analysis might exceed the available space or time resources of one’s computer (Asparouhov & Muthén, 2014), and as we did not encounter the latter issue, we believe that the MGCFA constituted a suitable framework for performing measurement invariance analyses in the present study. Third, the psychological construct of impulsivity was assessed by the 20-item short version of the UPPS-P Impulsive Behavior Scale (Billieux et al., 2012) rather than its parent form—the original 59-item UPPS-P Impulsive Behavior Scale (Cyders et al., 2007; Whiteside & Lynam, 2001)—despite the loss of content validity inherent to short forms (Smith et al., 2000). However, given that—in the context of the International Sex Survey (Bőthe et al., 2021)—participation consisted of completing online sociodemographic information questions and self-administered psychometric instruments totaling a maximum of 338 items, we believe that the loss of validity is justifiable in view of the considerable time saved. Last, detailed general limitations regarding the International Sex Survey are available from the Open Science Framework (https://osf.io/6kscb).

Taken together, the results of the present study substantiate the considerable corpus of research demonstrating the psychometric validity and reliability of the 20-item short version of the UPPS-P Impulsive Behavior Scale (Billieux et al., 2012), extending the latter psychometric instrument’s well-established relevance for measuring the psychological construct of impulsivity to a total of 26 countries, 13 languages, and three gender identities. Most notably, psychometric validity and reliability were evidenced across nine novel translations included in the present study (i.e., Croatian, English, German, Hebrew, Korean, Macedonian, Polish, Portuguese–Portugal, and Spanish–Latin American) and psychometric equivalence was evidenced across all three gender identities included in the present study (i.e., women, men, and gender-diverse individuals).

## Notes

1. At the time of publication of the International Sex Survey’s study protocol, 45 countries were included as collaborating countries. Although Egypt, Iran, Pakistan, and Romania were included in the study protocol as collaborating countries, these four countries did not receive timely ethical clearance from local ethics committees. In addition, although Chile was not included in the study protocol as a collaborating country, this country was subsequently included as a collaborating country and received timely ethical clearance from local ethics committees. Therefore, instead of the 45 countries included at the time of publication of the study protocol, 42 countries were included as collaborating countries in the present study. Detailed ethical information is available from the Open Science Framework (https://osf.io/e93kf) (https://osf.io/n3k2c/).

2. In examining measurement invariance hypotheses with ordered observed variables, imposing cross-group equality constraints on the model-implied non-null τ (i.e., item thresholds) unstandardized estimates is an identification condition for model-implied non-null ν (i.e., item intercepts) and θ (i.e., item residuals) unstandardized estimates (Wu & Estabrook, 2016).
